# Blind spot

**DOI:** 10.1038/s41390-021-01508-4

**Published:** 2021-04-06

**Authors:** Pontus M. Siren

**Affiliations:** Crassier, Switzerland

Despite six decades of intense effort, we do not have a common understanding of the underlying mechanism of sudden infant death syndrome (SIDS), and the question Doctor Samuel Fern posed in 1834: "what was the cause of death?", remains unanswered.^1^ The proliferation of SIDS hypotheses and the growing list of risk factors has muddled, rather than clarified our understanding, and the absence of robust animal models and pathological markers has frustrated our efforts to answer Fern’s question. However, there is also a collective blind spot that has contributed, perhaps decisively, to this frustration.

After decades of research, one would assume that all vital organs have been thoroughly investigated in the context of SIDS. Indeed, as of January 2021, from more than 12,500 SIDS publications, there are 2275 articles that focus on the heart, 999 on the central nervous system, 719 on the lungs, and 288 on the liver. These avenues of investigation have yielded important insights into the etiology of the syndrome. However, one critical organ has been ignored.

The diaphragm powers the vital respiratory pump, and its failure is a well-known terminal event in adults. Considering its importance and that SIDS most likely has a respiratory origin, one might assume that the diaphragm’s possible role in SIDS has been comprehensively investigated. However, only 59 PubMed articles on SIDS and diaphragm have been published over the past 50 years, and of these, seven relate to the SIDS-Critical Diaphragm Failure (SIDS-CDF) hypothesis.^2^

To understand this blind spot, we must consider if there are compelling reasons to exclude the diaphragm from SIDS research. I have found no such evidence, rather the opposite, and I encourage others to review the literature to determine if this exclusion is warranted.

For the past 10 years, I have repeatedly argued that the diaphragm must be investigated in the context of SIDS, if only to preclude it as a causative factor.^3^ Yet to this day, the diaphragm remains a collective blind spot.

## References

[1] Fern S (1834). Sudden and unexplained death of children. Lancet.

[2] Siren P (2017). SIDS-CDF hypothesis revisited: cause vs. contributing factors. Front. Neurol..

[3] Siren P, Siren M (2011). Critical diaphragm failure in sudden infant death syndrome. Ups. J. Med. Sci..

